# Full-time response of starch subjected to microwave heating

**DOI:** 10.1038/s41598-017-04331-2

**Published:** 2017-06-21

**Authors:** Daming Fan, Liyun Wang, Nana Zhang, Lei Xiong, Luelue Huang, Jianxin Zhao, Mingfu Wang, Hao Zhang

**Affiliations:** 1 State Key Laboratory of Food Science and Technology, Jiangnan University, 1800 Lihu Road, Wuxi, 214122 China; 2 School of Biological Sciences, The University of Hong Kong, Pokfulam Road, Hong Kong, China

## Abstract

The effect of non-ionizing microwave radiation on starch is due to a gelatinization temperature range that changes starch structure and properties. However, the changes in starch upon microwave heating are observable throughout the heating process. We compared the effects on starch heating by microwaves to the effects by rapid and regular conventional heating. Our results show that microwave heating promotes the rapid rearrangement of starch molecules at low temperatures; starch showed a stable dielectric response and a high dielectric constant. Microwave heating changed the Cole-Cole curve and the polarization of starch suspension at low temperatures. A marked transition at 2.45 GHz resulted in a double-polarization phenomenon. At temperatures below gelatinization, microwave-induced dielectric rearrangement and changes in the polarization characteristics of starch suspensions reduced the absorption properties; at temperatures above gelatinization, these characteristics became consistent with conventional heating. Throughout the heating process, microwaves change the electrical response and polarization characteristics of the starch at low temperatures, but on the macro level, there is no enhancement of the material’s microwave absorption properties. In contrast, with the warming process, the starch exhibited a “blocking effect”, and the absorption properties of the starch quickly returned to the level observed in conductive heating after gelatinization.

## Introduction

Microwave radiation is non-ionizing and changes the structural characteristics of materials during processing. It is one of the physical means used to modify the structural characteristics of starch. To promote the understanding and application of the physical effects of this process, Muzimbaranda^1^ *et al*. and Lewandowicz^2^ *et al*. reported on the interaction between microwave radiation and starch. These reports confirmed that microwave heating causes a rearrangement of intramolecular structures during gelatinization, thus altering the water absorption capacity, solubility, swelling ability, gelatinization characteristics, dehydration shrinkage behavior and rheological properties of starch^3–5^. We have long studied the relationship between gelatinization and the heating rate. The key factor that influences the physicochemical behavior of starch under microwave irradiation is the rapid heating effect; the thermophysical and structural properties are similar when conduction heating is carried out at the same rate as microwave heating^6–11^.

The effect of microwaves on materials differs from heat transfer in that microwave energy transfer is instantaneously stable and behaves like an excited electromagnetic wave. Some researchers have hypothesized that a microscopic response is instantly generated in material exposed to a microwave field; macroscopic changes may arise from an accumulation of microscopic responses or from the summation of microscopic responses together with other variables. Alternatively, a microscopic response may be too small to cause significant effects on the system. Traditional physical and chemical methods cannot verify or invalidate the above assumptions. We have found that electromagnetic and statistical methods can be used to preliminarily evaluate the characteristics of microwave absorption. In addition, we found that the absorption capability of starch differed in various gelatinization states. However, the causes for these differences and when they are generated are as yet unclear. As traditional methods cannot explain the above-mentioned process, it is necessary to use cross-disciplinary experimental and analytical methods to explore the electromagnetic nature of microwave radiation.

Polymers absorb microwaves by repositioning their dipoles in an applied electric field^12, 13^. The coupling efficiency between microwave radiation and polymeric material depends on the intensity, mobility, aggregation state and matrix state of the dipole^14^. The response of the dipole oscillation field consists of rotation and/or vibration, depending on the symmetry of the molecule; this molecular symmetry determines the degree of heat generation^15^. Based on this dipole mechanism, water is an important factor in the process of heating starch. Previous reports found that some low-wave properties of materials exhibited abnormal electromagnetic behavior in response to radiofrequency and microwave radiation after the materials were mixed with a strong dielectric material^16^. Starch is a carbohydrate polymer with a complex structure, composition and aggregation pattern. Pure liquid water absorbs microwave energy across a broad spectrum. Polymer matrix materials dispersed in water undergo different degrees of adsorption, binding and release upon heating; as a result, the material’s molecular polarization behavior changes and a power shift occurs in the dielectric response spectrum^17^.

Earlier studies have shown that the Cole-Cole model is superior to the macroscopic Debye model for describing the dielectric relaxation behavior of pure water^18^. The Cole-Cole model is usually used to study the Debye relaxation behavior of monomeric materials to determine the polarization behavior through the semicircular curve of the first quadrant; this constitutes a low-field detection method in which the Cole-Cole model describes the polarization behavior of the dielectric^16, 19^. Although researchers have used the Cole-Cole model to study the structural effects of high-field treatment on a single material, no study has yet applied this method to liquid mixed materials, especially for the study of liquid dispersions of food macromolecules.

We evaluated the difference between microwave heating and traditional heating and established a microwave heating (MW) model based on rapid conduction heating (RCV) temperature. Using traditional conduction heating (CV) as a control method, we investigated MW, RCV, and CV. We used these methods to investigate the change in the dielectric properties of starch in a microwave field. We discuss here the Debye polarization mechanism of the corresponding system, as well as the relationship between the variation and the absorption characteristics of the system.

## Materials and Methods

### Materials

Rice starch (protein content ≤ 0.23%, starch content ≥ 94.22%, amylose content 15.11%, particle size 5.52 ± 0.23 μm) was isolated and purified from fresh rice (QiuShouBao Rice Products Co., Ltd, Anhui, China).

A 30-g sample of fresh rice was steeped in 47 g distilled water for 18 h and ground into a slurry. The pH of the slurry was adjusted to 5.0 by adding 0.1 mol/L HCl; 0.04 g cellulase (XueMei enzymic preparation Sci. Co., Ltd., Wuxi, China) was then added to the slurry, which was allowed to react at 50 °C for 2 h. The pH was then adjusted to 7.0 by adding 0.1 mol/L NaOH, and 0.9 g neutral protease (0.8 Au/g, Novo Nordisk A/S, Denmark) was added and allowed to react at 50 °C for 4 h. The slurry was then centrifuged at 10,000 g for 30 min. The soft top layer was carefully removed and the starch layer was reslurried and washed nine times with deionized water. Butyl alcohol was added to the starch solution, stirring for 18 h to remove the fat content. The isolated starch was then dried in a freeze dryer, passed through a 150-μm sieve and stored in a dryer at room temperature until analyzed^8^.

### Heating conditions

#### Rapid conventional heating (RCV)

A thermostat (Thermo Fisher Scientific Co., Ltd., Massachusetts, USA) with an oil bath (200 °C) was used for rapid heating. The starch suspensions (100 g) with a concentration of 6% (w/w) were prepared with distilled water and heated to the desired temperature in the oil bath under constant agitation while monitoring the temperature online using an optical fiber probe (FISO Technologies Inc, Québec, Canada). The suspensions were then immediately placed into an ice bath to stop the gelatinization process. The treated samples were lyophilized and crushed to pass through a 75-μm sieve.

### Microwave heating (MW)

The MV experiment was conducted in the same manner as described above, except a microwave oven was used for heating. A 2450-MHz oven (Xianou Instrument Manufacturing Co., Ltd., Nanjing, China; MultiSYNTH, Milestone, Sorisole, Italy) with an output power of 1.2 kW was used to match the heating rate of the conventional heating with an oil bath. Multiple replications were performed in the preliminary stages to determine the power necessary to match the oil bath.

### Conventional heating (CV)

The CV experiment was conducted in the same manner as described above, except the sample mass was increased to 200 g and a hot plate was used for heating. Its measured heating rate was 0.13 °C/s.

### Dielectric measurements

The dielectric properties of the liquid system were measured using a vector network analyzer (E5071C, Agilent, Santa Clara, CA, USA) with an open-ended coaxial line, connected to a dielectric probe (85070E, Agilent, Santa Clara, CA, USA)^16, 19^. The probe was first calibrated using three materials (air, water of known temperature and metal), to ensure the accuracy of the measurements. Each sample was mixed to homogeneity before testing and was measured in triplicate.

### RL measurements

**Figure 1:**
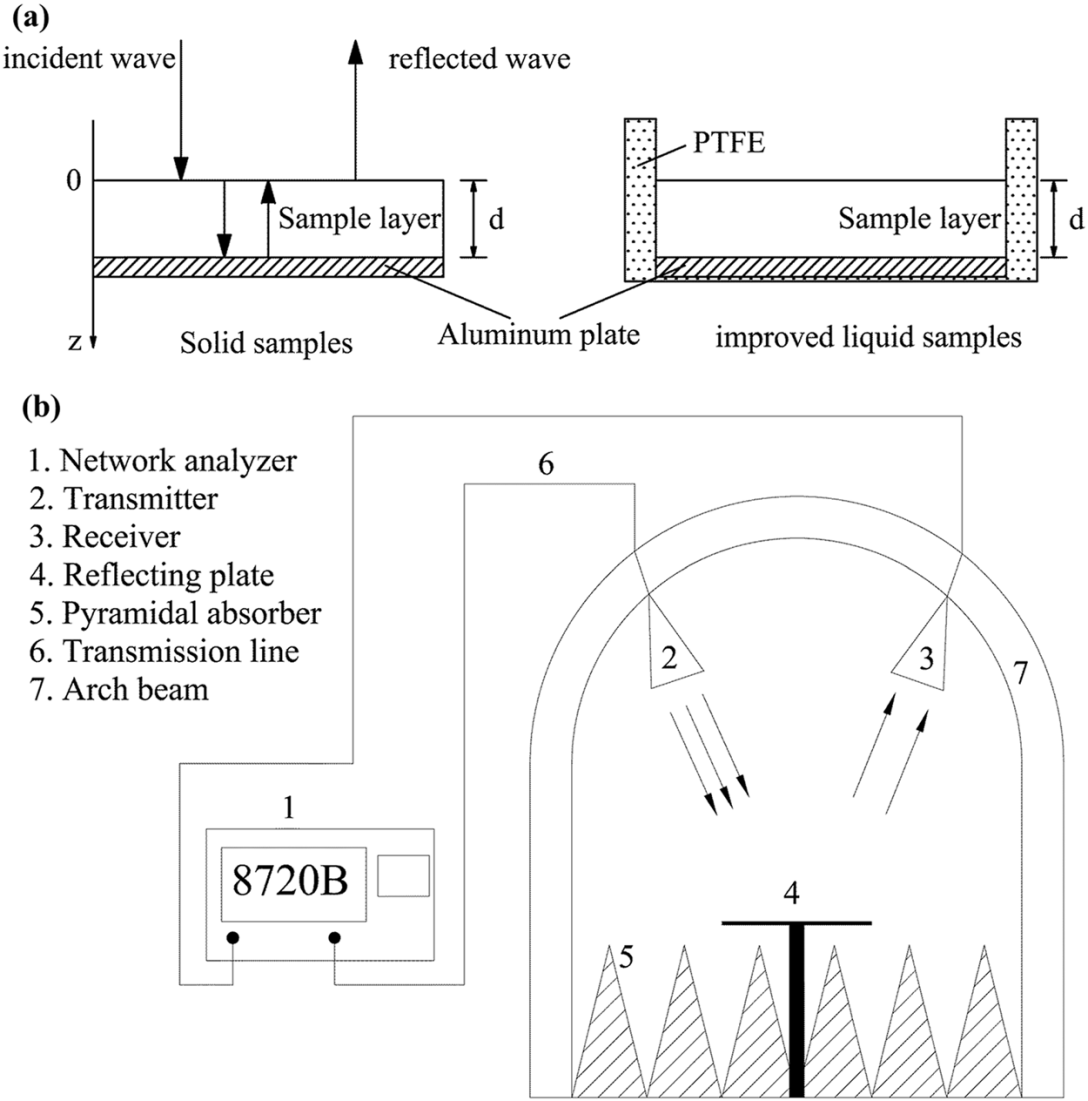
The arch method testing system for liquid samples. (**a**) The improved specialized container for liquid samples (**b**) The schematic diagram of the arch method testing system.

The reflection loss (RL) versus frequency for complex liquid materials was improved on the basis of the arch method by adding polytetrafluoroethylene (PTFE) as the wall material to the former aluminum plate, to make a specialized container (Fig. 1a). The testing system of the improved arch method included a vector network analyzer (HP 8720B, Hewlett Packard Co., Palo Alto, CA, USA) and standard horn antennas in an anechoic chamber as a function of frequency from 1.7 to 2.6 GHz (Fig. 1b)^12^.

### Data Availability

All data generated or analysed during this study are included in this published article.

## Results and Discussion

### Comparing the three heating methods

**Figure 2:**
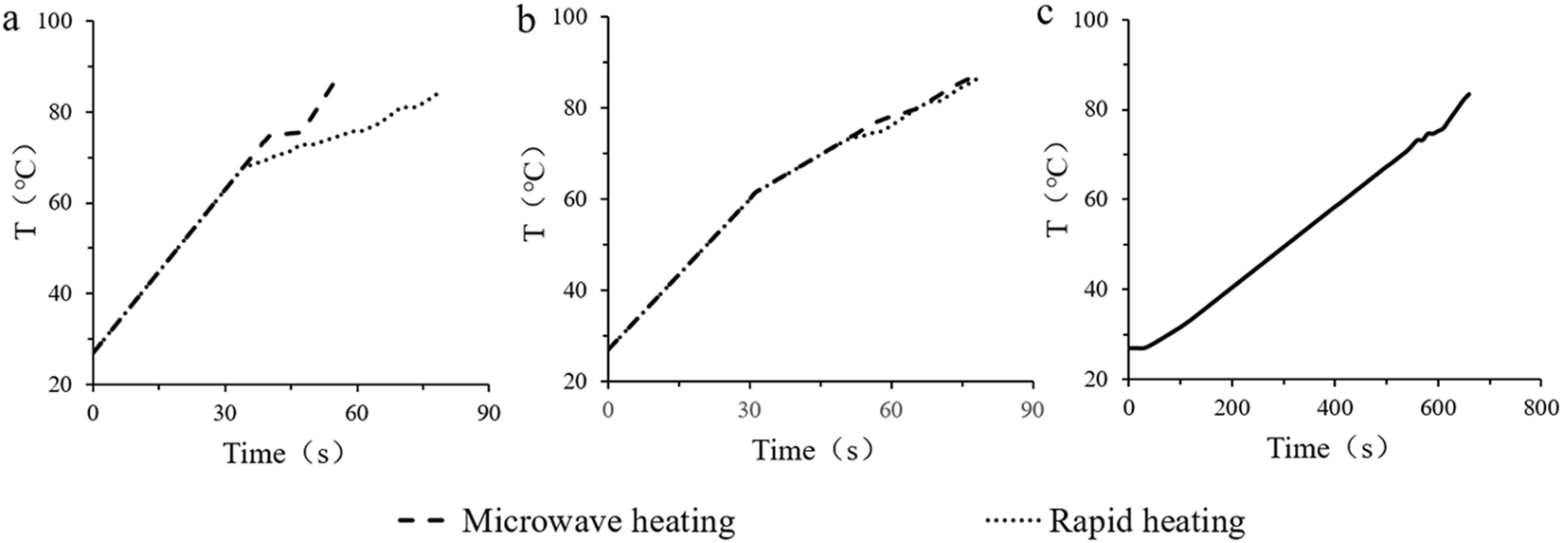
Temperature curves of the samples treated with three different heating methods. (**a**) Temperature curves of the samples treated with microwave heating (1200 W) and rapid conventional heating. (**b**) Temperature curves of the samples heated using microwave heating (1200 W × 32 s, 600 W × 20 s, 300 W × 14 s, 600 W × 12 s) and rapid conventional heating. (**c**) Temperature curves of the samples treated using conventional heating.

Previously we studied the gelatinization of rice starch heated by microwave heating, comparing the time-temperature profiles for MW and RCV up to 85 °C. Above 70 °C the heating rate of RCV decreased, whereas that of MV increased. Hence, we had to use the multi-stage heating program of MV to match the profile of RCV^6, 7^. However, we paid attention to the subgelatinization levels in this work, in which the highest heating temperature was 70 °C. The multiple correlation coefficient obtained from the function corrcoef in MATLAB was 0.9998. Therefore, the RCV model fitted well with the microwave processing (Fig. 2). As observed, the heating rate of MV and RCV was 1.12 °C/s. The temperature curve of the samples treated with the CV heating method (with a heating rate of 0.05 °C/s) is shown in Fig. 2c.

### Dielectric properties of starch under microwave irradiation

There are many models for studying the dielectric properties of a mixed system. For example, the formulas of Onsager, Kirkwood and Clausius-Mossotti^20–22^ can be used to calculate the permittivity of a mixed system; most of these systems are calculated in an electrostatic field. Considering that the boundary conditions and the model system must be perfect, the calculated equivalent permittivity is significantly different from the measured value^23^.

1 $$\frac{{\varepsilon }_{eff}-{\varepsilon }_{e}}{{\varepsilon }_{i}+{\varepsilon }_{e}+v({\varepsilon }_{eff}-{\varepsilon }_{e})}=\alpha \frac{{\varepsilon }_{i}-{\varepsilon }_{e}}{{\varepsilon }_{i}+{\varepsilon }_{eff}+v({\varepsilon }_{eff}-{\varepsilon }_{e})}$$

Where, *ε*_*ε*_ *, ε*_*i*_, *ε*_*eff*_ are the dielectric constants of the substrate medium, mixed medium and mixture, respectively; α is the mixing ratio; and ν is the standard parameter suitable for different mixing rules. For example, *v* = 0 denotes the Maxwell Garnet formula, *v* = 2 denotes the Bruggeman formula and *v* = 3 denotes the coherent potential approximation formula. We can conclude from Equation (1) that the calculated values of the equivalent permittivity are generally between the mixed components; this often proves that the real part of the dielectric constant of the mixed system is larger than that of any component^24^. The macroscopic thermophysical properties of starch in hot and humid conditions change greatly. Ndife *et al*. showed that the heat treatment process influenced the main components of a starch-based mixed system, resulting in a considerable change in dielectric properties^25^. Therefore, the classical mixed formula is not suitable for describing the dielectric properties of mixed systems. In this experiment, based on the reaction model established, the frequency-scanning curves of a mixed system under the three heating conditions were measured, thus elucidating the influence of the heating process on electromagnetic properties.

**Figure 3:**
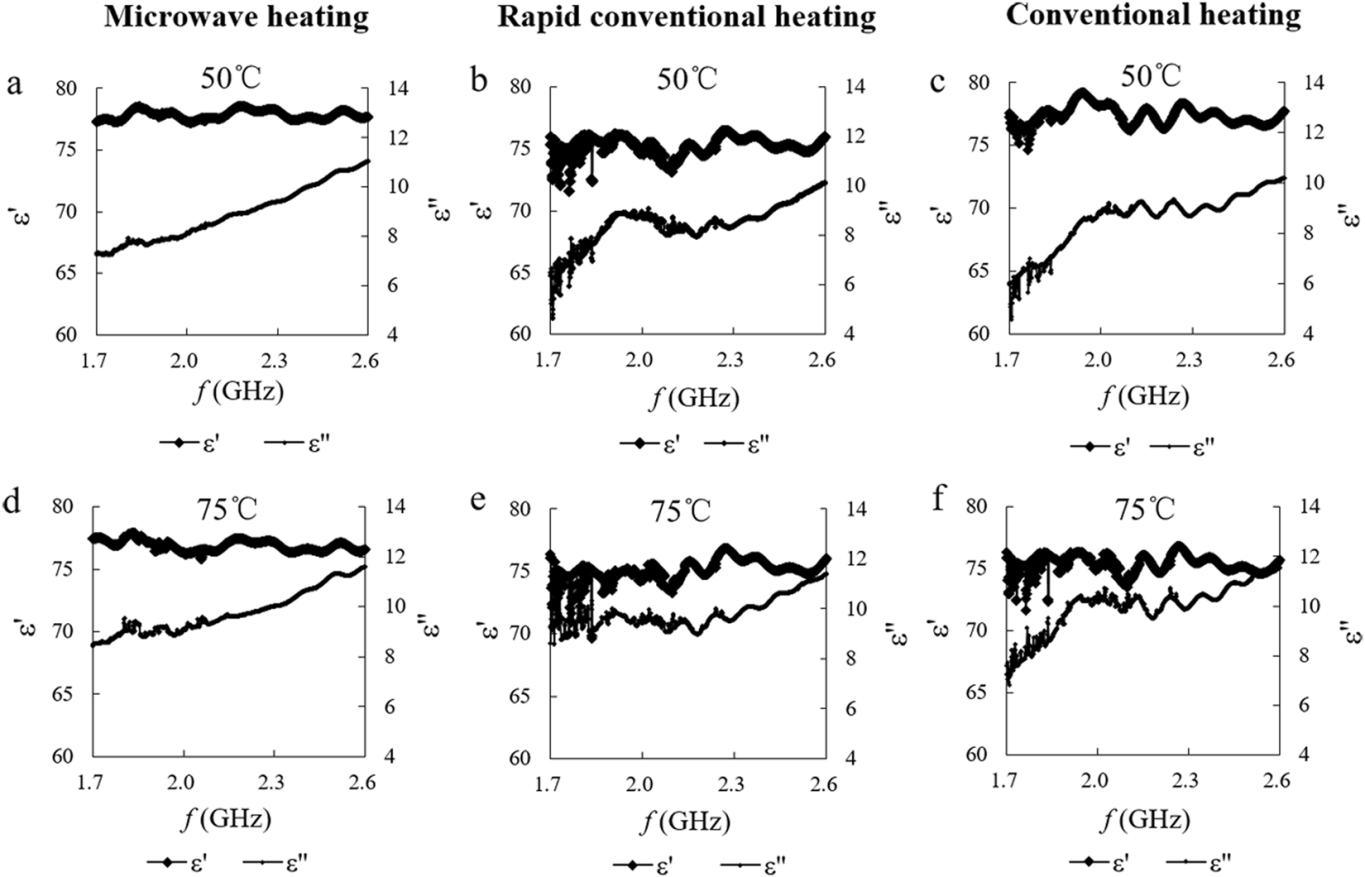
Frequency-scanning curves of dielectric properties of starch using different heating protocols under characteristic temperatures.

Figure 3 shows the change in the dielectric properties of a starch suspension during a frequency sweep of 1.7–2.6 GHz. Comparing the frequency-scanning curves of three kinds of heating method before and after gelatinization, we found that the gelatinization process did not affect the wave profile of the starch–water system; the correspondence of each method was strong. ε′ slightly changed and ε′′ increased after heating treatment. Comparison among the three heating methods yielded the following results: (i) frequency sweep curves of fast transmission and traditional heating were very similar, (ii) the signal-to-noise ratio of the MW sample was at a minimum before and after the gelatinization and (iii) the change in the dielectric properties was most stable in the range of 1.7–2.6 GHz. Thus, the microwave treatment induced the normalization of the dipole moments of the main components in the aqueous system, resulting in a relatively stable polarization relaxation behavior. Based on the dielectric scanning curve, we investigated the change of dielectric properties with temperature under 2.45 GHz.

**Figure 4:**
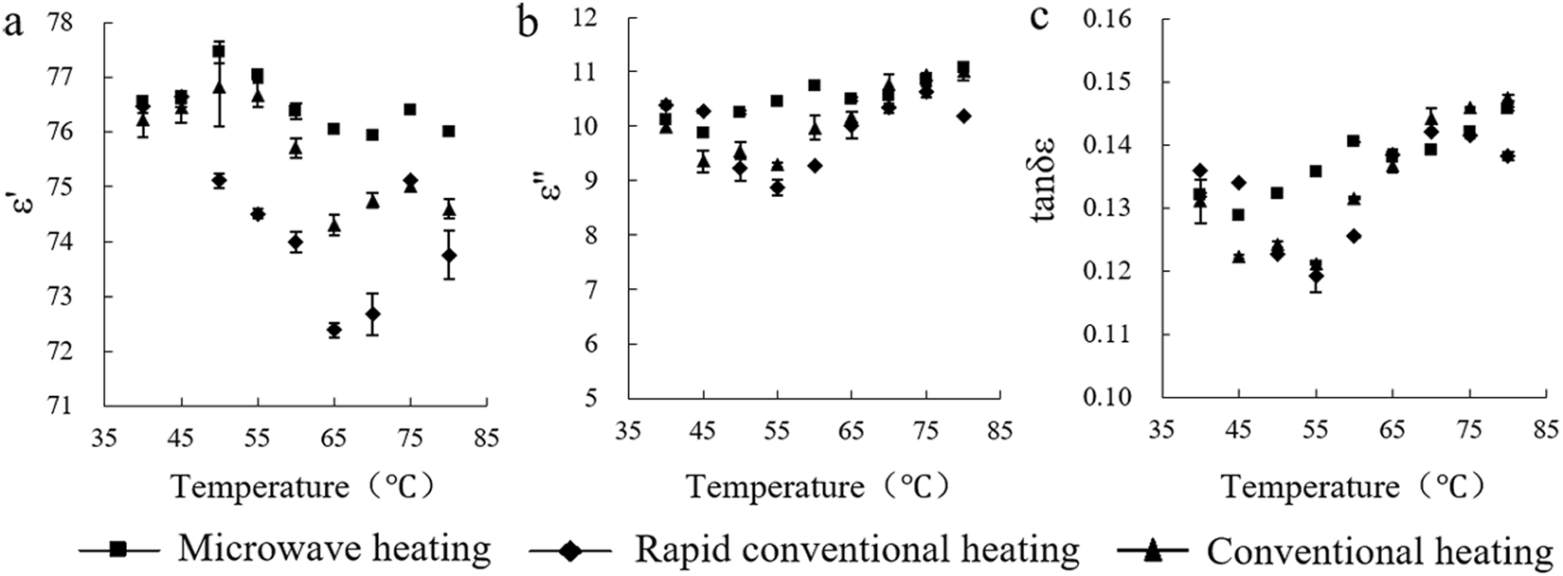
Curves of starch dielectric properties versus temperature at 2.45 GHz. (**a**) Permittivity (**b**) Dielectric loss (**c**) Dielectric loss tangent.

Figure 4 shows that starch exhibited a certain degree of volatility when exposed to these three heating methods. Microwave-heated samples showed the strongest dielectric response, characterized by a relatively stable and low sum of ε′ (Fig. 4a), ε″ (Fig. 4b) and tanδε (Fig. 4c), especially in the temperature range of 45 °C to 65 °C; the dielectric response of rapidly heated samples decreased in the range of 50 °C to 70 °C. Thus, the microwave treatment showed a relatively stable dielectric response in the low range of temperature. Microwave treatment affected the orientation polarization of starch, which was rapid enough to provide the further conditions for the interaction of high temperature and electromagnetic waves. The rapid heating effect was larger in starch. Compared with traditional heating, there was no time for the polar structure of starch to adapt to the effects of heat treatment, leading to a threshold-stage mixture decrease. At the end of the gelatinization process, the uniformity of the system increased and the dielectric response recovered.

With an increase in temperature, numerous variables were influential. Examples are the orientation of containment between the hydroxyl polar groups of the starch molecule, dipole rotation degree, Brownian motion and the molecular thermal motion effect. There was real-time interference with dipole orientation that resulted in weakened intermolecular interactions, reorientation and rapid destruction at high temperatures. Thus, the change of the hydrogen bond affected the high-frequency dispersion friction^26–28^, resulting in fluctuations of ε′ and ε″ in the mixed solution.

An analysis of the starch dielectric constant based on the hydrogen bond dynamics during the heat treatment revealed the breakdown of ordered and disordered (amorphous and amorphous) intermolecular hydrogen bonding in starch. In addition, swelling of the starch granules resulted in the expansion of bonded hydroxyl in the granule to form a hydrogen bond with H_2_O, enhancing the tetrahedral structure of molecular H_2_O. The non-polar glucose ring ensured the formation of a hydrophobic hydration layer. Therefore, two categories of H_2_O molecules exist in the system: H_2_O molecules that form strong hydrogen bonds with starch, and the continuous-volume H_2_O and the H_2_O molecules that migrate alone. In this process, two H_2_O molecules are linked to an oxygen atom on one hydroxyl group in the starch glucose unit, and the hydrogen atom on the hydroxyl group is linked to the oxygen atom on the H_2_O molecule, resulting in the formation of the intermolecular chain structure^29^. Hydrogen bonds between starch and H_2_O were stronger than those of H_2_O molecules but weaker than internal hydrogen bonds of starch granules. Therefore, the different heat treatments affected hydrogen bonds in the starch suspension state, changing the high-frequency friction of the starch suspension, which led to the fluctuation of ε″.

At a microwave frequency of 2.45 GHz, a specific phenomenon was observed during the dielectric constant test of the starch emulsion: the heating mixture system’s ε″ was larger than any of the components. We examined next the behavior of the relaxation in the Debye model to investigate the causes of this specific phenomenon in the mixed system.

### Debye polarization mechanism of starch under microwave field

The most basic physical concept of the dielectric effect is polarization, the offset of the positive and negative charge centers in the media. The establishment and change of polarization, known as the dielectric relaxation, occurs gradually over time as one equilibrium state transforms to another. In essence, the dielectric relaxation reflects the energy exchange and the related energy loss of microscopic particles during polarization^30^. Here we introduce the concept of complex permittivity. The real aspect of complex permittivity reflects the ability of the dielectric effect to store energy in the polarization process, whereas the imaginary aspect describes the energy dissipation during the polarization process. In the present study, three polarization mechanisms of the medium were ion polarization, electron polarization and electric dipole moment polarization. Ion polarization and electron polarization in the microwave frequency range are relatively weak, whereas they are more obvious in the infrared frequency range^31, 32^. Therefore, we assume that the dipole moment polarization in the gelatinization process leads to a change in the dielectric loss of starch. In the process of polarization, microwave energy turned into heat, resulting in loss.

The Debye equation introduces the concept of relaxation time, which describes dielectric relaxation as follows:

2 $${\rm{\varepsilon }}^{\prime} ={{\rm{\varepsilon }}}_{\infty }+\frac{{{\rm{\varepsilon }}}_{{\rm{s}}}-{{\rm{\varepsilon }}}_{\infty }}{1+{{\rm{\omega }}}^{2}{{\rm{\tau }}}^{2}}$$

3 $${\rm{\varepsilon }}^{\prime\prime} =\frac{({{\rm{\varepsilon }}}_{{\rm{s}}}-{{\rm{\varepsilon }}}_{\infty }){\rm{\omega }}{\rm{\tau }}}{1+{{\rm{\omega }}}^{2}{{\rm{\tau }}}^{2}}$$

4 $$\tan \,{\rm{\delta }}=\frac{({{\rm{\varepsilon }}}_{{\rm{s}}}-{{\rm{\varepsilon }}}_{\infty }){\rm{\omega }}{\rm{\tau }}}{{{\rm{\varepsilon }}}_{{\rm{s}}}+{{\rm{\varepsilon }}}_{\infty }{{\rm{\omega }}}^{2}{{\rm{\tau }}}^{2}}$$

Where, ε_s_ and ε_∞_ are static and optical frequency permittivities, respectively, *ω* is angular frequency, ω = 2π*f* and *τ* is relaxation time.

Eliminating *ωτ* in Equation (4) provides a semicircle equation called the Cole-Cole circle.

5 $${[{\rm{\varepsilon }}^{\prime} -\frac{1}{2}({{\rm{\varepsilon }}}_{{\rm{s}}}+{{\rm{\varepsilon }}}_{\infty })]}^{2}+{({\rm{\varepsilon }}^{\prime\prime} )}^{2}=\frac{1}{4}{({{\rm{\varepsilon }}}_{{\rm{s}}}-{{\rm{\varepsilon }}}_{\infty })}^{2}$$

The Cole-Cole circle is useful in experimental studies. If the experimental data of the real and imaginary parts of the dielectric constant form a semicircular arc, the material adheres to the ideal Debye equation, and only one major polarization mechanism exists. If the experimental data form two or more semicircular arcs, then two or more major polarization mechanisms are active. If the experimental data form a semicircular arc and “tail” (right), the material is a high-loss material that exhibits an interface polarization mechanism.

In contrast, if we rewrite the Debye Equation (6) as shown in Equation (7), we can make a straight line as (ε′, ε″/ω) and (ε′, *ω*ε″) according to the test results of the real and imaginary part of dielectric constant under the limited frequency; thus, we can obtain parameters *τ*, ε_∞_ and ε_s_ according to the slope and intercept.

6 $${\rm{\varepsilon }}^{\prime} =\frac{1}{{\rm{\tau }}}(\frac{{\rm{\varepsilon }}^{\prime\prime} }{{\rm{\omega }}})+{{\rm{\varepsilon }}}_{\infty }$$

7 $${\rm{\varepsilon }}^{\prime} =-{\rm{\tau }}({\rm{\omega }}{\rm{\varepsilon }}^{\prime\prime} )+{{\rm{\varepsilon }}}_{{\rm{s}}}$$

For a dielectric effect that deviates from the Debye relaxation, the modified Debye equation from the Cole-Cole model is suitable, as shown in Equation (8):

8 $${\rm{\varepsilon }}={\rm{\varepsilon }}^{\prime} -j{\rm{\varepsilon }}^{\prime\prime} ={{\rm{\varepsilon }}}_{\infty }+\frac{{{\rm{\varepsilon }}}_{{\rm{s}}}-{{\rm{\varepsilon }}}_{\infty }}{{(1-j{\rm{\omega }}{\rm{\tau }})}^{1-{\rm{\alpha }}}}$$

Where, τ_a_ is the average relaxation time, *α* is a positive number between zero and 1, and *α* can be used to measure the applicability of the Debye parameters. The larger *α* is, the more the dielectric deviates from the Debye relaxation.

The Debye equation is a phenomenological theory that describes the complex energy exchange behavior of microscopic particles in the polarization process as attributed to relaxation time. Relaxation time τ is a function not only of composition and temperature, but also of the structure and microstructure of the material. However, the effect of the inhomogeneous microstructure of the material on the dielectric relaxation can be included in the value of *α* in the modified Debye equation.

Finally, note that when the electric field frequency is very high (ω→∞), the Debye equation contains a ε′ → ε∞, ε → 0 function. In fact, the dielectric loss is still in the optical frequency (optical absorption), which is ignored in the Debye relaxation theory. Therefore, the Debye equation is limited because it is only suitable for a frequency range far below the optical frequency range^33^.

The simplified Cole-Cole equation is ${\rm{\varepsilon }}^{\prime} =\frac{1}{{\rm{\tau }}}(\frac{{\rm{\varepsilon }}^{\prime\prime} }{{\rm{\omega }}})+{{\rm{\varepsilon }}}_{1}$. If there is only one mechanism for dielectric loss, the curve should be a straight line in which the real part of complex permittivity ε′ is the abscissa, B_0_ (v)is the ordinate, and 1/2πf is the slope corresponding to the polarization frequency of Debye $\,{f}_{r}=|1/\tau |$. The universal microwave frequency range used for heating is 2.45 GHz, and the application tolerance in practical research, production and life is ± 0.05 GHz. Thus, we studied the influence of microwave energy on starch in the range of 2.40 to 2.50 GHz.

**Figure 5:**
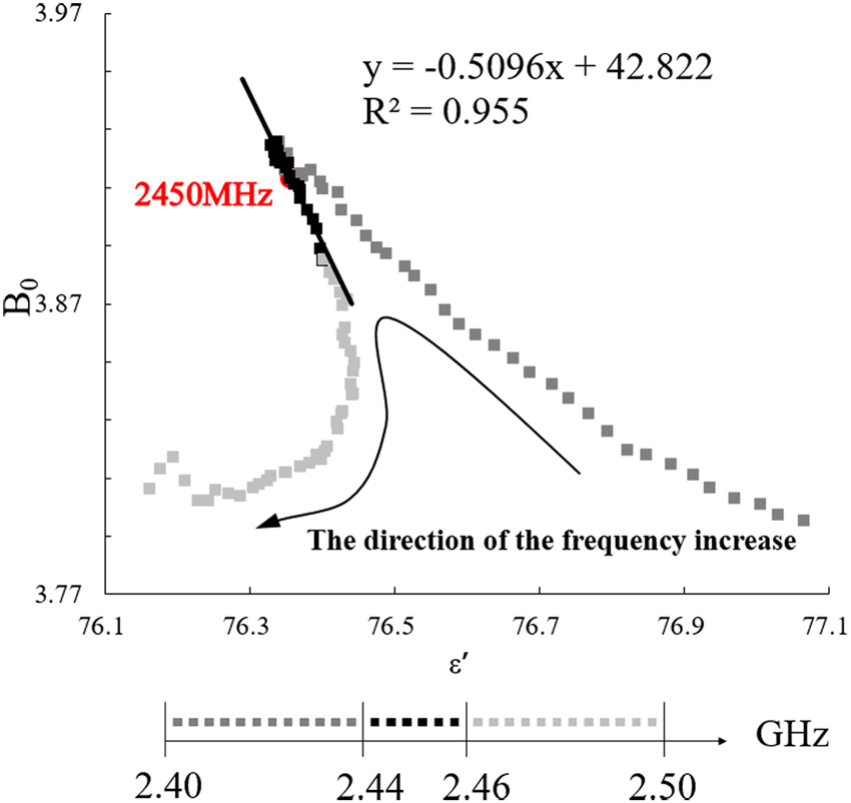
Change curve of starch–water mixtures with B_0_ (${{\rm{B}}}_{0}={\rm{\varepsilon }}^{\prime\prime} /f$) varying with ε′ at room temperature (25 °C).

Figure 5 shows the relationship between a starch suspension’s variation B_0_ (${{\rm{B}}}_{0}={\rm{\varepsilon }}^{\prime\prime} /f$) and the real part of the complex permittivity ε′ at room temperature. From the figure, the material B_0_ ~ε′ curve shows a continuous and relatively smooth trend, which means that the whole process corresponds to a continuous relaxation time spectrum rather than to a single relaxation time. Thus, the relaxation time is not a single time even for the extremely uniform particle size in a monodispersed system^34^. Compared with an interval of 2.46–2.50 GHz, the interval of 2.40–2.44 GHz exhibited a similar geometric change process and a smaller geometric change. An apparent B_0_ value transition occurred at approximately 2.5 GHz, but the interval of 2.45 ± 0.01 GHz was in a stable range that can be fitted by a straight line. The calculated response of the Debye polarization was ${f}_{{\rm{r}}}=3.202\,{\rm{GHz}}$.

Starch has both a reducing end and a nonreducing end; its positive and negative charge centers do not coincide. In terms of the long chain of starch molecules, these two centers are very close; therefore, starch is electrically neutral and is a non-polar molecular dielectric. The positive and negative charges of water molecules do not coincide and thus form an electric dipole; Thus, water is a polar molecular dielectric. In a conventional state, the orientation of the electric dipole within the water molecules tends to be consistent because of irregular thermal motion; therefore, the synthetic dipole moment is not zero.

It is generally believed that a polarization frequency corresponds to an electric dipole moment polarization mechanism, which may be due to the polarization of the system interface or a defect of the polarization. Surface polarization often occurs at the interface of heterogeneous materials with different electromagnetic properties. A starch suspension is a homogeneous mixture of materials whose minimal electron-scattering characteristics result from an outer layer of electrons caused by an ion-conductive current accumulating in the starch particles and at the water interface. In addition, the hybrid structure of water molecules causes them to interact strongly with the outer layer of starch granules at the interface. This interaction leads to a change in the density of states of electron spin polarization and an asymmetric charge distribution at the interface. Note that there is no Debye polarization frequency corresponding to each polarization, meaning that the electromagnetic field under the polarization-induced ultrastructural response still needs to be further clarified.

**Figure 6:**
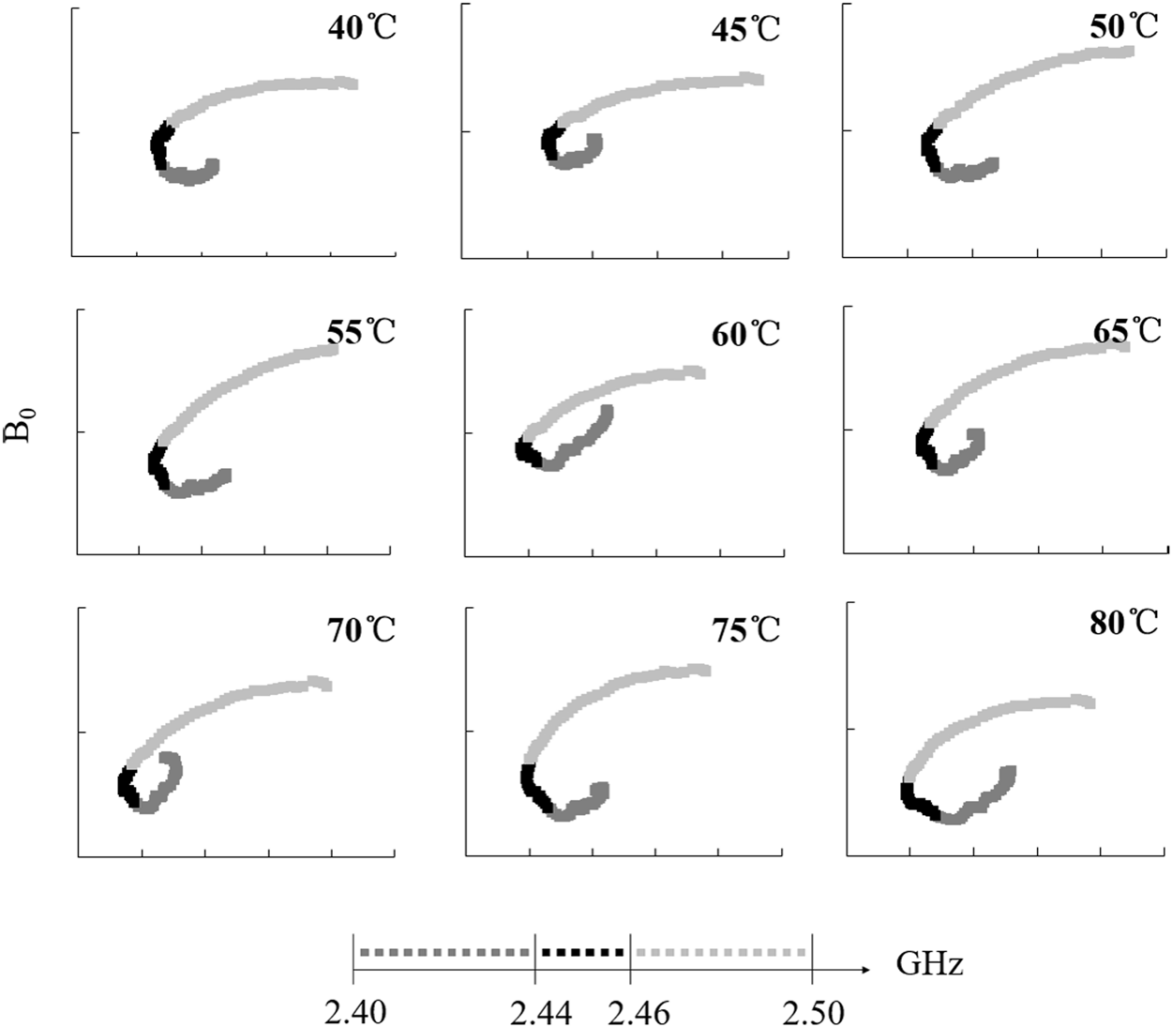
Change curve of starch–water mixtures B_0_ (${{\rm{B}}}_{0}={\rm{\varepsilon }}^{\prime\prime} /f$) varying with ε′ with microwave heating.

If the starch suspension is placed in a microwave field, the charged particles can change their distribution state because of action of the electromagnetic field force. From a macro-perspective, changes in the system’s polarization, magnetization and conduction are observed. Figure 6 shows that the overall shape of the B_0_~ε′ curve of samples was consistent under microwave heating at different temperatures; a continuous and smooth trend was observed in the short interval 2.40–2.44 GHz with small geometric changes. Large geometric changes were observed in the long 2.46–2.50 GHz interval. A turning point was observed at 2.45 ± 0.01 GHz; there were two fitting straight lines at each temperature point. The fitting equations and the calculation of the polarization is shown in Table 1. With an increase in temperature, the polarization frequency I showed a rapid low frequency transfer except for the lowest frequency at 60 °C. We think that this mechanism is easily affected by the temperature; a higher polarization frequency of 4.794 GHz was evident in comparison with the normal temperature sample at 40 °C. Polarization frequency II is relatively stable, indicating that such a mechanism does not have a strong temperature response after microwave treatment. The periodic distribution of charge at the starch/water interface may be the source of interfacial polarization under the action of electromagnetic waves^35^.

**Table 1:** The fitting equations of Cole-Cole curves and polarization frequency for starch–water mixtures treated using microwave heating.

Temperature (°C)	Equation I	Polarization frequency I (GHz)	Equation II	Polarization frequency II (GHz)
40	y = −0.7630x + 62.621	4.794	y = 0.3363x–21.651	2.113
45	y = −0.4734x + 40.225	2.974	y = 0.2468x–14.837	1.551
50	y = −0.4580x + 39.665	2.878	y = 0.4158x–28.021	2.612
55	y = −0.3548x + 31.605	2.229	y = 0.5906x–41.229	3.711
60	y = −0.1032x + 12.266	0.648	y = 0.4604x–30.784	2.893
65	y = −0.2928x + 26.548	1.840	y = 0.5992x–41.272	3.765
70	y = −0.2611x + 24.147	1.640	y = 0.5614x–38.319	3.527
75	y = −0.2422x + 22.937	1.522	y = 0.4794x–32.183	3.012
80	y = −0.1177x + 13.470	0.740	y = −0.5777x + 48.428	3.630

**Figure 7:**
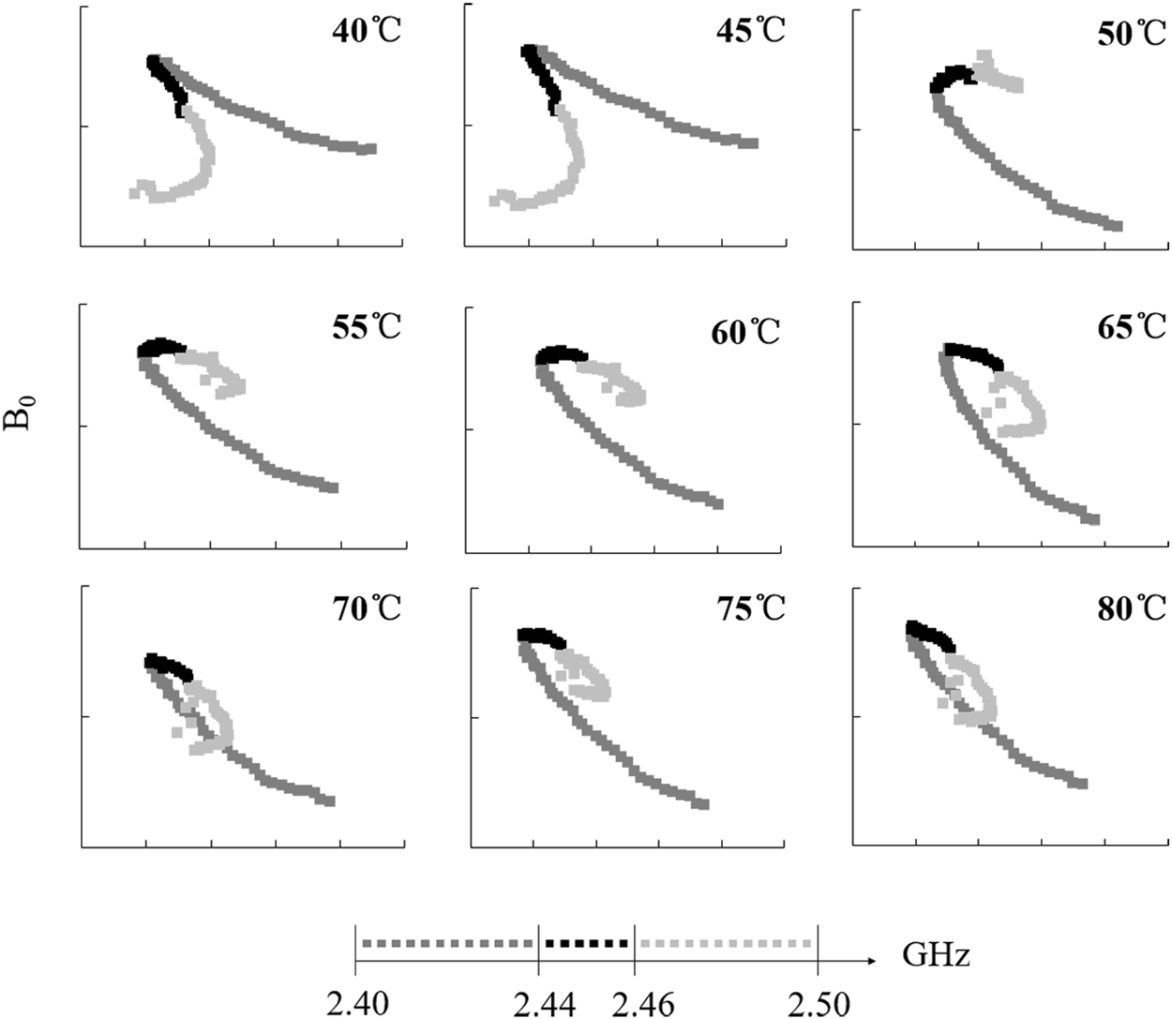
Change curve of starch–water mixtures B_0_ (${{\rm{B}}}_{0}={\rm{\varepsilon }}^{\prime\prime} /f$) varying with ε′ with rapid conventional heating.

Figure 7 shows that the B_0_~ε′ curves were divided into two types by using the rapid thermal conduction method to treat samples at different temperatures. The morphology of samples at 40 °C and 45 °C was similar to that shown in Fig. 5, showing a continuous smooth trend. The sample at temperatures above 50 °C exhibited a long interval and extensive geometric changes in the range of 2.40–2.44 GHz. We observed a short interval of 2.46–2.50 GHz with few geometric changes and an obvious B_0_ transition close to 2.44 GHz. The range of 2.45 ± 0.01 GHz was a stable range that was fitted by a straight line. The fitting equations and calculations of the polarization are shown in Table 2. With an increase of temperature, the polarization frequency shifted to a low frequency. The frequency of 40 °C was 2.63 GHz. At 50 °C, the frequency moved to below 1 GHz. The lowest frequency appeared close to 60 °C. The same mechanism was also affected largely by temperature.

**Table 2:** The fitting equations of Cole-Cole curves and polarization frequency for starch–water mixtures treated using rapid conventional heating.

Temperature (°C)	Equation	Polarization frequency (GHz)	Temperature (°C)	Equation	Polarization frequency (GHz)
40	y = −0.4190x + 36.304	2.633	65	y = −0.0882x + 10.463	0.554
45	y = −0.3210x + 28.788	2.017	70	y = −0.1008x + 11.545	0.633
50	y = 0.1233x–5.498	0.775	75	y = −0.0537x + 8.372	0.337
55	y = 0.0082x + 3.017	0.052	80	y = −0.1203x + 13.033	0.756
60	y = −0.0036x + 4.057	0.023			

**Figure 8:**
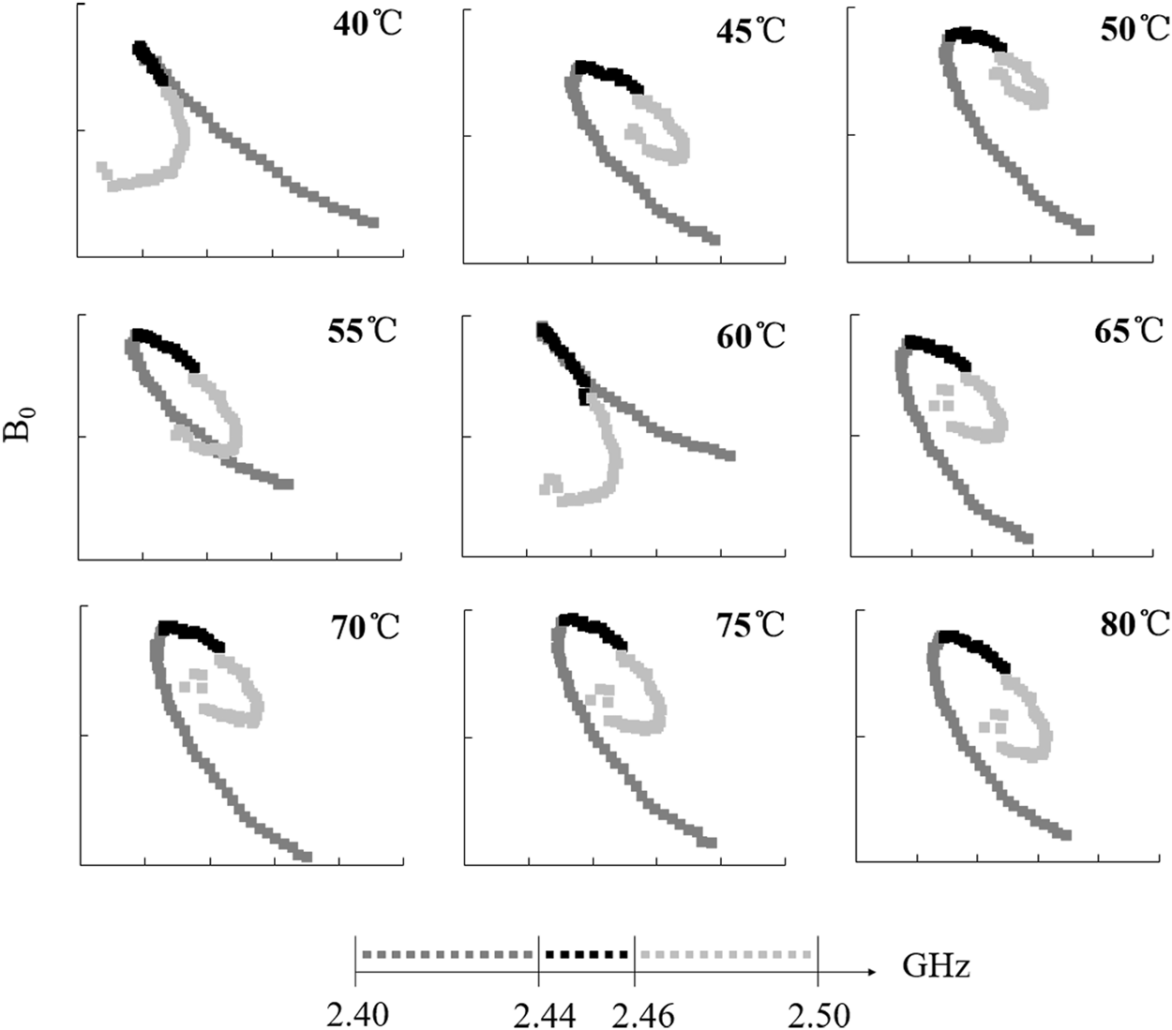
Change curve of starch–water mixtures B_0_ (${{\rm{B}}}_{0}={\rm{\varepsilon }}^{\prime\prime} /f$) varying with ε′ treated with conventional heating.

Figure 8 shows that the overall shapes of B_0_~ε′ curves were divided into two types by using the traditional CV method to treat samples at different temperatures. The morphology of samples at 40 °C was similar to that in Fig. 7, exhibiting a continuous smooth trend. The sample at temperatures above 45 °C exhibited a long interval and large geometric changes in the range of 2.40–2.44 GHz. We observed a short interval of 2.46–2.50 GHz with few geometric changes and an obvious B_0_ transition close to 2.44 GHz. The range 2.45 ± 0.01 GHz was stable and could be fitted by a straight line. The fitting equations and the calculation of the polarization are shown in Table 3. With increasing temperature, the polarization frequency was 2.633 GHz at 40 °C, and the frequency was below 1 GHz at 45 °C. The lowest frequency appeared close to 50 °C. This mechanism is also probably affected by temperature.

**Table 3:** The fitting equations of Cole-Cole curves and polarization frequencies for starch–water mixtures treated using conventional heating.

Temperature (°C)	Equation	Polarization frequency (GHz)	Temperature (°C)	Equation	Polarization frequency (GHz)
40	y = −0.3573x + 31.160	2.245	65	y = −0.1124x + 12.498	0.706
45	y = −0.0959x + 11.149	0.602	70	y = −0.0860x + 10.829	0.540
50	y = −0.0579x + 8.341	0.364	75	y = −0.1145x + 13.056	0.719
55	y = −0.1506x + 15.336	0.946	80	y = −0.1271x + 13.968	0.798
60	y = −0.1469x + 15.114	0.923			

Horizontal comparison of the three methods revealed considerable differences between MW-treated samples and the other two samples. The observed prominent differences in curve trend and progress exerted a strong influence on the polarization characteristics of starch suspensions, leading to a dual-polarization phenomenon in the system. The trend of polarization frequency I of samples heated with MW samples was similar to the temperature change trend in the RCV and CV, possibly because of interfacial polarization. In other words, starch particles swell in the water phase, and the particle state changes considerably with temperature, thus affecting the interface of the positive and negative charge distribution. In this mechanism, the microwave process below 40 °C strongly increased the material polarization frequency. The higher the frequency, the greater the conductivity of the medium. When the transmission attenuation was faster, the transmission distance was shorter. In other words, microwave treatment can improve the polarization properties of materials at low temperatures. The polarized frequency II of MW was 2.45 GHz. After microwave treatment, the microwave dipole moment response of the system was stable, and interference was caused by the weak electric field formed by a microwave-induced electric dipole moment. The dual dielectric polarization of a microwave-induced starch suspension enhances the ability of microparticles to absorb electromagnetic waves.

### Effect of microwave heating on microwave-absorbing properties of starch

Previous studies have shown that rice starch suspensions produce a rapid response in a microwave field. Microwaves promote a dual-polarization phenomenon in rice starch suspensions. The increase in the microscopic mechanism probably changes the suspension’s ability to absorb microwaves. To clarify this issue, it is necessary to further study the macroscopic microwave absorption properties of the materials when they are treated with microwave radiation at different temperatures.

**Figure 9:**
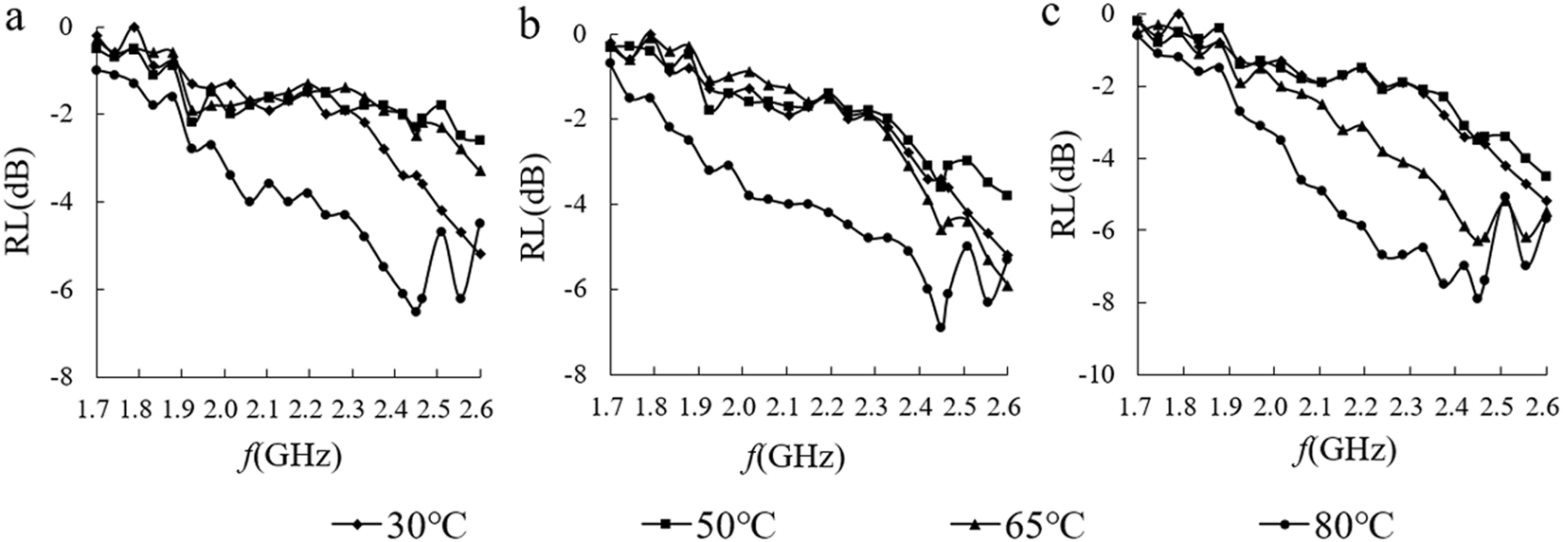
RL curves of starch–water mixtures treated by three heating methods. (**a**) Microwave heating (**b**) Rapid conventional heating (**c**) Conventional heating.

**Figure 10:**
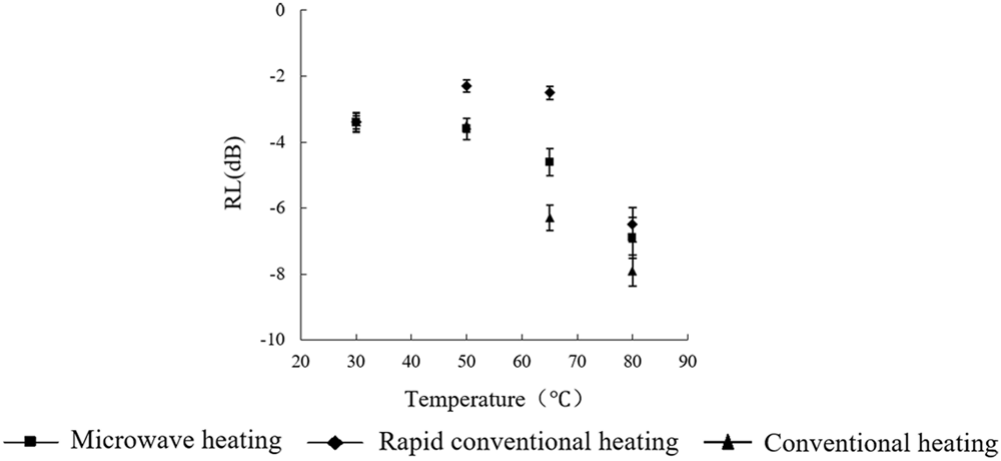
RL curves at 2.45 GHz of starch–water mixtures treated using three heating methods.

Figure 9 shows the relationship between the electromagnetic wave absorption properties of the three kinds of heat-treated samples and frequency in the temperature-rise period. In the longitudinal comparison, the microwave absorption law for the three methods in the frequency-sweeping process is similar; that is, the whole curve showed a small absorption value between 30 °C and 65 °C and a large absorption value at 80 °C. In the range of 1.7–2.6 GHz, the maximum absorption and frequency turning point at each temperature were observed at 2.45 GHz. The frequency-scanning curves of the three methods are similar. The absorption values of the whole scanning process with MW were similar to the values observed in the RCV method but were below the CV sample, especially at 80 °C and 65 °C. At a frequency of 2.45 GHz, as shown in Fig. 10, the MW samples exhibited a decrease in the absorption value and a strong absorption characteristic at 65°C or higher. RCV and CV samples also showed an increase of absorption value; an outstanding absorption ability occurring at 65 °C was mainly due to the gelatinization of starch.

The above results indicate a marked reduction in the microwave absorption properties of water upon mixing the original starch with water at room temperature (25 °C). This reduction is due mostly to the absence of particulate (not hydrated) starch in a strong electric field. The charged particles cannot break through internal forces between water molecules. Because of the limit of the electrostatic charges on water molecules on the interface layer, at low starch concentrations the water-starch system generates a strong electromagnetic absorption interference. However, with an increase in temperature, starch granules absorb water and swell, resulting in a breakdown of lamellar starch structure and damage to entangled starch chains. During the hydration process, the positive and negative charges of the starch molecules do not coincide, so that the microscopic particles form an electric dipole, begin to exhibit polarity and produce polarization. In addition, as the degree of gelatinization of the system increases, there is considerable enhancement of the absorption characteristics of the materials.

After microwave treatment, the microwave absorption properties of the material did not increase under the experimental conditions, but showed a certain blocking effect in the heating process. We suggest three reasons for this blocking effect. First, combined with impact of the microwave on dielectric properties of the materials, the results showed that ε′, ε″ and tanδε of microwave-treated samples were higher than they were with the other two heating protocols before the gelatinization temperature was reached to cause the numerical increase in dielectric properties. Regarding electromagnetic characteristics, the greater the difference in dielectric constant and permeability, the easier it was to generate a blocking effect that influenced the absorption of electromagnetic waves. However, the starch aqueous permeability was about 1 with no marked magnetic effects; therefore, the microwave heating process amplifies the dielectric permeability difference and reduces microwave absorption. Second, although the electromagnetic loss of microwave-treated material is relatively increased, an impedance matching process of a two-phase system in the electromagnetic wave incident process was changed because the system exhibited a rearrangement of charge based on the material characteristics in the heating process. Therefore, microwave energy incident at the surface and interface was greatly reflected or offset, and that energy was not completely absorbed by the material. Third, combined with the effect of microwaves on the material Debye polarization mechanism, the results explain that although the microwave treatment at low temperatures changes the polarization characteristics of the material close to the frequency of 2.45 GHz, increasing the polarization process of the material, the dual-polarization characteristics of microwave treatment is related to the intrinsic polar moment of the polar molecules and the microelectronic polarization or atomic polarization^36^. This experiment revealed an ion polarization contribution^37^, thus, double-polarization characteristics resulted from the combined action of multiple starch structures that antagonize the polarization process to a certain extent. Further research should help to elucidate these effects.

## Conclusions

With microwave heating, starch exhibits a relatively stable electromagnetic response before and after gelatinization. The starch suspension rearranges at low temperatures, inducing standardization of the electric dipole moment in its main components and forming a more stable polarization relaxation behavior; in addition, we observe more prominent and stable dielectric properties. At the microcosmic level, microwave heating has a marked effect on the Cole-Cole reduction curve of starch suspensions, changing the original polarization and producing a double-polarization phenomenon that affects the microwave absorption based on the polarization mechanism. At the macroscopic level, microwave heating hinders the microwave-absorbing properties of starch suspensions below the gelatinization temperature; this is consistent with the conduction heating method following gelatinization.

We could observe effects of microwaves on starch and the production of starch suspensions throughout the heating process. During the early stage of heating, microwaves immediately affected the dielectric response and the polarization mechanism of the starch. During the middle and late stages of heating, the microwave-absorbing properties under a microwave field markedly changed with the thermal conversion process. This provides a powerful basis for further explanation of microwave thermal runaway.
